# One‐Year Outcomes After Intravenous Recombinant Tissue Plasminogen Activator for Ischemic Stroke: A Real‐World Study

**DOI:** 10.1111/cns.70543

**Published:** 2025-08-07

**Authors:** Rui Xue, Xiaoxian Gong, Yuhui Huang, Wansi Zhong, Haidi Jin, Zhicai Chen, Yi Chen, Shenqiang Yan, Haitao Hu, Changzheng Yuan, Min Lou

**Affiliations:** ^1^ Department of Neurology The Second Affiliated Hospital, Zhejiang University School of Medicine Hangzhou China; ^2^ State Key Laboratory of Transvascular Implantation Devices Hangzhou China; ^3^ School of Public Health The Second Affiliated Hospital, Zhejiang University School of Medicine Hangzhou China; ^4^ Department of Nutrition Harvard T.H. Chan School of Public Health Boston Massachusetts USA

**Keywords:** acute ischemic stroke, all‐cause mortality, chinese, intravenous recombinant tissue plasminogen activator, long‐term outcomes, thrombolysis

## Abstract

**Background:**

The longer‐term benefits of intravenous recombinant tissue plasminogen activator (IV rt‐PA) in Chinese acute ischemic stroke (AIS) patients remain lacking. We aimed to evaluate the 1‐year clinical outcomes after IV rt‐PA for Chinese AIS patients in a real‐world setting.

**Methods:**

Based on a prospective multicenter stroke registry in China, we analyzed the data of patients with AIS (aged ≥ 18 years) who arrived at hospital within 4.5 h of symptom onset. Participants were from 80 stroke centers in China between January 2017 and March 2020. IV rt‐PA‐treated patients were propensity score‐matched (1:1) by baseline characteristics with non‐reperfusion patients. Primary outcome was 1‐year all‐cause mortality. Secondary outcomes included 1‐year functional outcomes.

**Results:**

Participants were mostly male (59.9%), with a mean age of 70.2 years. One‐year all‐cause mortality was similar between the two groups (11.1% vs. 12.2%; HR, 0.90 [95% CI: 0.78–1.05], *p* = 0.183). At 1 year, the IV rt‐PA group had a higher proportion of functional independence (modified Rankin Scale [mRS] 0–2: 70.9% vs. 66.4%; OR, 1.25 [95% CI, 1.12–1.39]) and favorable outcome (mRS 0–1: 59.5% vs. 54.6%; OR, 1.23 [95% CI, 1.11–1.36]) compared to the non‐reperfusion group (both *p* < 0.001). A lower proportion of severe disability/death was also observed in the IV rt‐PA group versus the non‐reperfusion group (mRS 5–6: 15.9% vs. 20.3%; OR, 0.73 [95% CI, 0.64–0.83]) (all *p* < 0.001).

**Conclusions:**

IV rt‐PA treatment in Chinese AIS patients eligible for thrombolysis was associated with improved 1‐year functional outcomes despite having similar mortality to those who did not receive any reperfusion treatments.

**Trial Registration:** This study is registered on https://clinicaltrials.gov; Unique identifier: NCT0539533.

## Graphical abstract

Chinese patients with acute ischemic stroke treated with intravenous recombinant tissue plasminogen activator (IV rt‐PA) within 4.5 h of symptom onset had similar rates of all‐cause mortality 1‐year post‐AIS compared with non‐reperfusion (11.1% vs. 12.2%, respectively). At 1 year, compared with non‐reperfusion, IV rt‐PA was associated with significantly higher rates of functional independence and favorable outcomes, and a lower rate of severe disability/death.

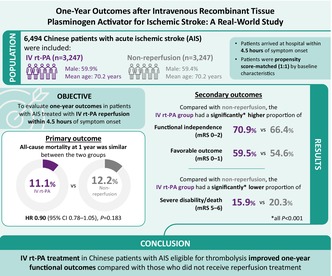

## 1 Introduction

The recent absolute number of deaths due to ischemic stroke worldwide has risen to approximately 3.29 million in 2019 and has been estimated to increase by almost 50% to 4.9 million by 2030 [1]. In China, the reported rates of stroke and associated burden are higher than those of other high‐income countries. Despite the observed downward trend of stroke‐related case fatalities [2], the risk remains high, with a 12‐month fatality rate of 6.0% in Chinese patients experiencing a first‐ever ischemic stroke and a 12‐month disability rate (defined as modified Rankin Scale [mRS] score 3–5) of 14.2% [3].

Thrombolytic therapy with intravenous recombinant tissue plasminogen activator (IV rt‐PA) has been shown to improve functional outcomes and mortality risk at 90 days when administered within 4.5 h of symptom onset in patients with acute ischemic stroke (AIS) [4, 5], including in Asian and Chinese patients [6, 7, 8, 9, 10]. IV rt‐PA improved functional outcomes at 1 year without increasing the mortality risk at 3 months [11]. However, the treatment effect on long‐term mortality beyond 6 months has yet to be confirmed [12]. An earlier randomized controlled trial did not find a survival benefit with IV rt‐PA at 1 year compared with patients who were not treated with IV rt‐PA [13]; while there have been subsequent analyses of non‐Asian data from large real‐world registries, the results regarding the effect of IV rt‐PA on 1‐year or longer‐term (up to 3 years) mortality risk were inconsistent [14, 15, 16, 17, 18].

This study aimed to evaluate the 1‐year mortality and functional outcomes of IV rt‐PA delivered within 4.5 h of symptom onset using propensity score matching of Chinese patients with AIS from a real‐world database.

## 2 Methods

### 2.1 Study Design and Population

This study (ClinicalTrials.gov number: NCT05395338) was a retrospective analysis based on a prospective multicenter stroke registry in China, the Computer‐based Online Database of Acute Stroke Patients for Stroke Management Quality Evaluation (CASE‐II). Patients with AIS (aged ≥ 18 years) who arrived at the hospital within 4.5 h of symptom onset from 80 stroke centers between January 2017 and March 2020 were included in the study. Key exclusion criteria were contraindication (except age) to IV rt‐PA treatment; missing key data (i.e., age, gender, baseline National Institutes of Health Stroke Scale [NIHSS], time of symptom onset, time of hospital arrival/admission, whether they received IV thrombolysis, time of IV rt‐PA treatment); received thrombolytic agents other than rt‐PA; received endovascular treatment; or received IV rt‐PA after 4.5 h of symptom onset. Eligible patients were divided into two groups: 1) those who received IV rt‐PA within 4.5 h of symptom onset: the IV rt‐PA group, and 2) those who did not receive any reperfusion treatments (primarily due to family/patient refusal): the non‐reperfusion group.

### 2.2 Data Collection and Management

CASE‐II was initiated in 2016 and designed to monitor the current status of regional stroke care in Zhejiang [19]. The database included de‐identified patient demographic information, baseline clinical characteristics, and indicators related to diagnosis and treatment during hospitalization and follow‐up after discharge (a telephone contact follow‐up was conducted at 1‐year post‐discharge). To ensure reliability, all follow‐ups were conducted by a third‐party team of mRS certified neurologists using a standardized script adapted from the mRS Telephone Questionnaire. All telephone interviews were recorded and traceable for subsequent verification. Assessors were blinded to treatment allocation or the baseline clinical and radiographic characteristics of the individual patient. All assessors underwent structured training before conducting follow‐ups. During the first follow‐up, assessments were conducted by two assessors to evaluate consistency. Subsequently, all assessors received retraining every 3 months to ensure the accuracy and consistency of the follow‐up process. All patient data were entered by local trained registrars using standardized protocols, and the overall process of case registration, monitoring of the data quality, and inquiry and correction of erroneous data was managed and supervised by a steering committee.

### 2.3 Study Outcomes

Primary outcome was 1 year (defined as 12 ± 1 months) all‐cause mortality (mRS = 6). Secondary outcomes were functional independence (mRS 0–2), favorable clinical outcomes (mRS 0–1), severe disability or death (mRS 5–6), and the distribution of mRS scores at 1 year. Safety outcome was any intracranial hemorrhage (defined by the European Cooperative Acute Stroke Study [ECASS] criteria) during hospitalization.

### 2.4 Sample Size

Based on the assumption of 12% and 15% for 1‐year mortality in the IV rt‐PA and non‐reperfusion cohorts, respectively, with a power of 90% at a two‐sided, 5% significance level, a sample size of approximately 5400 patients was required to observe 756 events to detect a difference.

### 2.5 Statistical Analysis

All continuous data were assessed for normality using the Kolmogorov–Smirnov test. Continuous variables were described as mean (standard deviation [SD]) or median (interquartile range [IQR]) and categorical variables as numbers and percentages. The IV rt‐PA and non‐reperfusion cohorts were propensity score‐matched 1:1 based on age, gender, baseline NIHSS, pre‐stroke mRS, medical insurance status, smoking status, hospital level, comorbidities (e.g., diabetes, coronary artery disease, atrial fibrillation, prior stroke/transient ischemic attack, and hypertension), co‐medications (e.g., antiplatelet, lipid lowering, and oral anticoagulation medications), and time from symptom onset to hospital admission. The method of nearest neighbor matching (a maximum caliper width of 0.2 of the SD of the logit of the propensity score) was used [20]. Missing data for pre‐stroke mRS scores were imputed with the median. We calculated the absolute standardized difference (ASD) to compare baseline characteristics between the IV rt‐PA and non‐reperfusion groups. Propensity score matching and multivariate analysis were used to address potential sources of bias; baseline characteristics that were not sufficiently balanced (ASD ≥ 0.1) after propensity score matching were included in an appropriate multivariate model to adjust for the differences. Propensity score matching was performed using the MatchIt package in R 4.0.5; all other analyses were conducted with SAS 9.4.

The primary outcome was analyzed using Kaplan–Meier curves and Cox regression. Secondary outcomes were analyzed using conditional logistic regression; distribution of mRS at 1 year was analyzed using ordinal logistic regression, and the common odds ratio (OR) was reported. A sensitivity analysis was performed to include patients who received endovascular treatment and IV rt‐PA for the primary and secondary outcomes. Exploratory analyses were performed for the primary and secondary outcomes in the following subgroups: baseline NIHSS score (0–4, 5–10, 11–15, 16–21, and ≥ 22), time from symptom onset to treatment (≤ 90, 91–180, and 181–270 min), age (18–80 and > 80 years), and rt‐PA dose (low dose of approximately 0.6 mg/kg and standard dose of 0.9 mg/kg). Based on the observed results from planned analyses, a post hoc analysis was performed to evaluate all‐cause mortality at < 150 days and ≥ 150 days after the onset of AIS. To further assess the robustness of our findings, we performed a post hoc analysis with multiple imputations for missing data on 1‐year mRS scores and re‐evaluated the primary and secondary outcomes in the propensity score‐matched IV rt‐PA and non‐reperfusion cohorts. To simultaneously achieve the representativeness of results and balanced characteristics between groups, we also performed a propensity score weighting that included the non‐matched cohort as a sensitivity analysis.

### 2.6 Ethics

This study was approved by the Second Affiliated Hospital of Zhejiang University Institutional Review Board. The study was designed and performed in accordance with the study protocol, the Declaration of Helsinki, the International Conference on Harmonization Harmonized Tripartite Guidelines for Good Clinical Practice, and applicable national and local regulations.

## 3 Results

### 3.1 Patient Demographics and Baseline Characteristics

Of the final 12,551 eligible patients with AIS, 8014 patients received IV rt‐PA treatment and 4537 patients did not receive reperfusion treatment (Figure S1). Baseline characteristics of the whole study population are summarized in Table S1. Baseline characteristics of patients with and without data on all‐cause mortality at 1 year are shown in Table S2.

After propensity score matching, a total of 6494 patients were included for analysis, of whom 3247 patients treated with IV rt‐PA were matched against 3247 patients who did not receive reperfusion (Figure S1). There were no significant differences in the baseline patient demographics and clinical characteristics of the IV rt‐PA cohort versus the non‐reperfusion cohort (males: 59.9% vs. 59.4%; mean ± SD age: 70.2 ± 11.6 vs. 70.2 ± 12.9 years; median (IQR) NIHSS score: 4 (2–8) vs. 3 (1–7); prior mRS score 0–1: 97.0% vs. 97.1%; mean ± SD time from symptom onset to hospital admission: 132.9 ± 56.4 vs. 137.1 ± 68.6 min) (Table 1). The majority of the IV rt‐PA cohort (92.0%) received the standard dose of rt‐PA at baseline (Table 1).

**TABLE 1:** Baseline Characteristics of the Propensity Score‐Matched IV rt‐PA and Non‐Reperfusion Cohorts.

Variables	IV rt‐PA (*N* = 3247)	Non‐reperfusion (*N* = 3247)	Absolute standardized difference
Age, mean (SD), years	70.2 (11.6)	70.2 (12.9)	0.002
Male, *n* (%)	1944 (59.9)	1929 (59.4)	0.009
Medical insurance status, *n* (%)			0.050
Urban employee basic medical insurance	955 (29.4)	961 (29.6)	
Urban resident basic medical insurance	1038 (32.0)	1048 (32.3)	
New rural cooperative medical insurance	657 (20.2)	697 (21.5)	
Other insurance	250 (7.7)	232 (7.1)	
No insurance	347 (10.7)	309 (9.5)	
Smoking status, *n* (%)			0.015
Never	2232 (68.7)	2255 (69.4)	
Former	213 (6.6)	207 (6.4)	
Current	802 (24.7)	785 (24.2)	
Prior mRS score 0–1, *n* (%)	3151 (97.0)	3154 (97.1)	0.005
NIHSS score, median (IQR)	4 (2–8)	3 (1–7)	0.057
Time from symptom onset to hospital admission, mean (SD), min	132.9 (56.4)	137.1 (68.6)	0.067
Time from symptom onset to treatment, mean (SD), min	184.5 (54.2)	NA	NA
Time from hospital admission to treatment, mean (SD), min	52.6 (26.3)	NA	NA
rt‐PA dosage, *n* (%)			NA
Standard dosage	2573 (92.0)	NA	
Low dosage	223 (8.0)	NA	
Comorbidities, *n* (%)			
Diabetes	559 (17.2)	561 (17.3)	0.002
Coronary artery disease	258 (7.9)	246 (7.6)	0.014
Atrial fibrillation	442 (13.6)	450 (13.9)	0.007
Prior stroke/transient ischemic attack	606 (18.7)	623 (19.2)	0.013
Hypertension	2143 (66.0)	2099 (64.6)	0.028
Co‐medication, *n* (%)			
Antiplatelet	514 (15.8)	506 (15.6)	0.007
Oral anticoagulation	63 (1.9)	73 (2.2)	0.022
Lipid lowering	340 (10.5)	340 (10.5)	< 0.001
Hospital level, *n* (%)			0.007
Grade 2	766 (23.6)	775 (23.9)	
Grade 3	2481 (76.4)	2472 (76.1)	

*Note:* Absolute standardized difference < 0.1 (not significant) for all baseline characteristics.

Abbreviations: IQR, interquartile range; IV, intravenous; mRS, modified Rankin Scale; NA, not applicable; NIHSS, National Institutes of Health Stroke Scale; rt‐PA, recombinant tissue plasminogen activator; SD, standard deviation.

### 3.2 Outcomes

**FIGURE 1:**
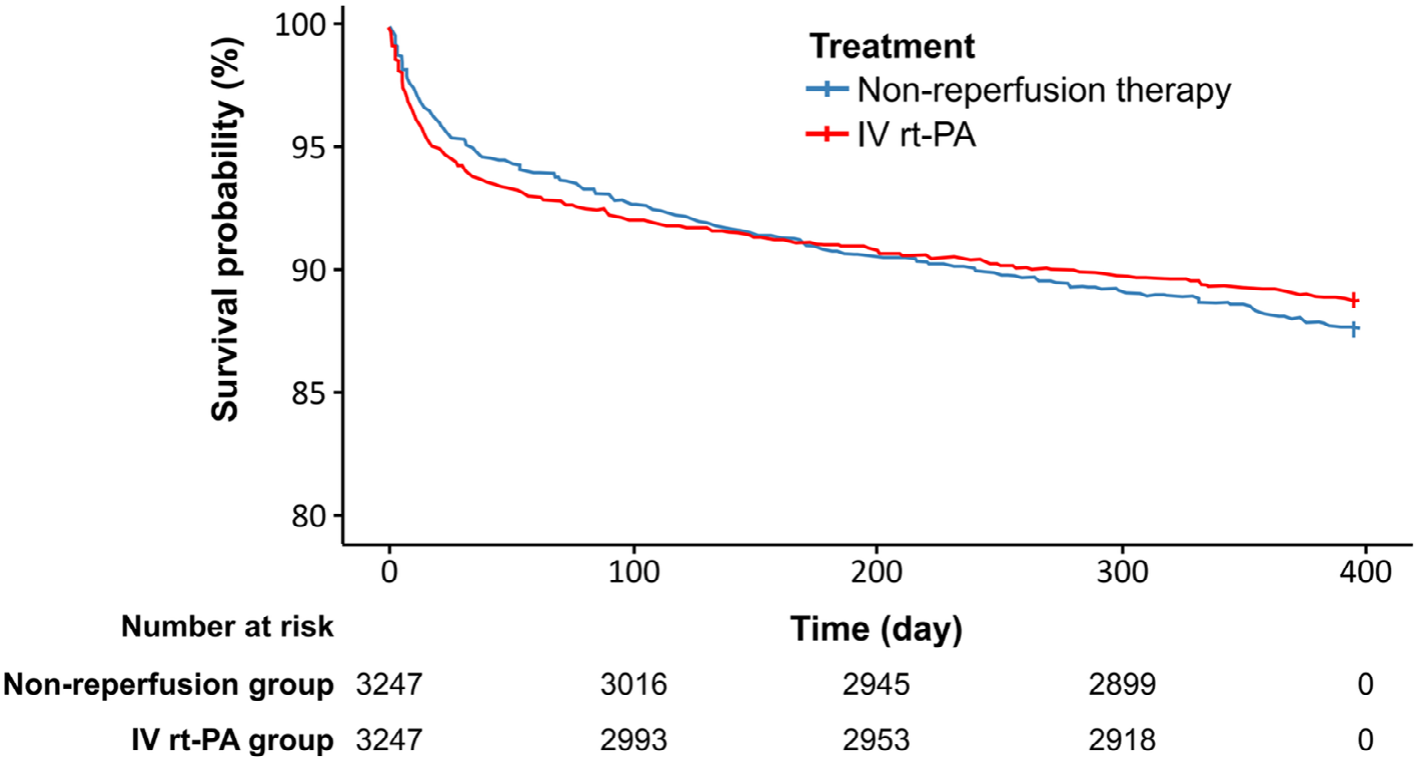
Kaplan–Meier curve for all‐cause mortality within 1 year. IV, intravenous; rt‐PA, recombinant tissue plasminogen activator.

**FIGURE 2:**
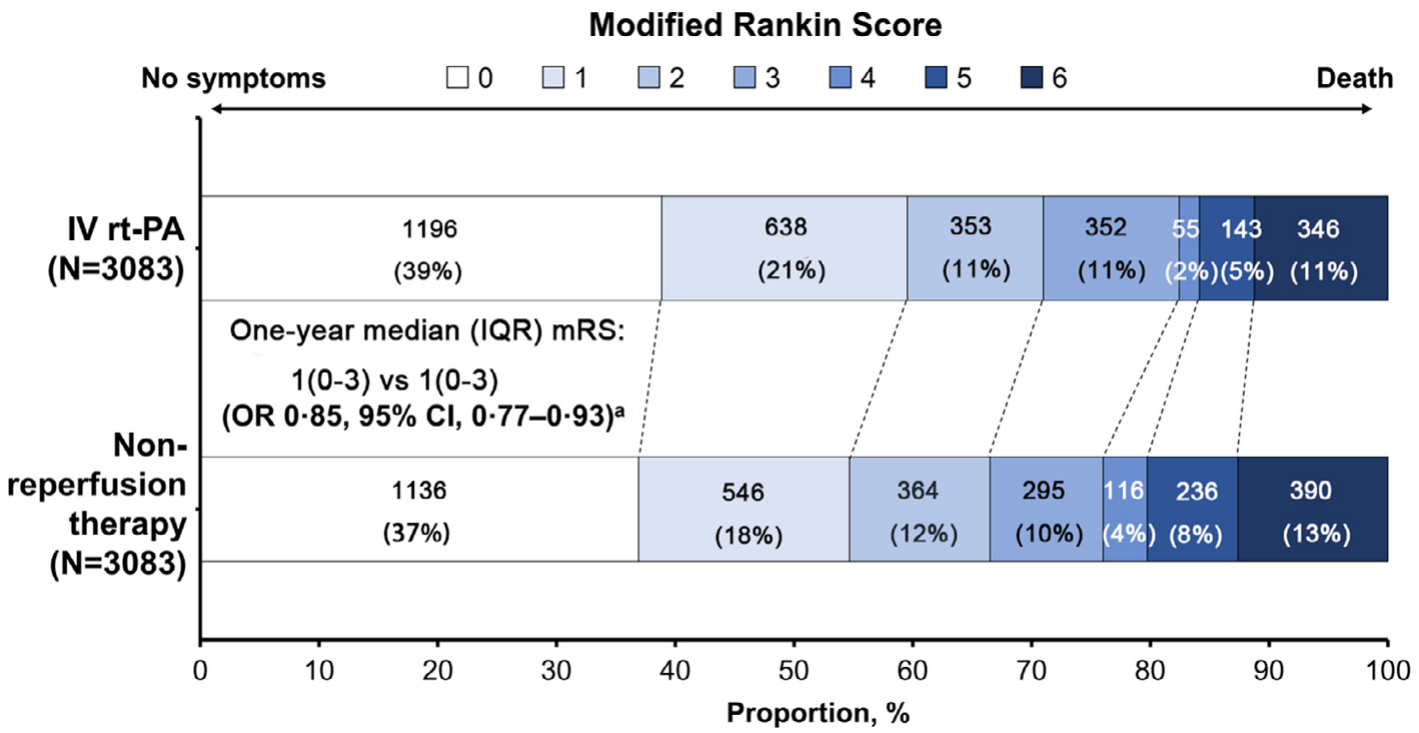
Distribution of mRS scores in the propensity score‐matched IV rt‐PA and non‐reperfusion cohorts at 1 year. Data from patients with available mRS scores at 1 year (*N* = 3083). ^a^ *p* < 0.001, derived from ordinal logistic regression. CI, confidence interval; IV, intravenous; mRS, modified Rankin Scale; OR, common odds ratio; rt‐PA, recombinant tissue plasminogen activator; IQR, interquartile range.

Kaplan–Meier estimates showed that the 1‐year all‐cause mortality was similar in the IV rt‐PA cohort versus the non‐reperfusion cohort (11.1% vs. 12.2%, hazard ratio [HR] 0.90 [95% confidence interval (CI), 0.78–1.05]; *p* = 0.183) (Figure 1; Table 2). There were no significant differences in any intracranial hemorrhage during hospitalization (8.3% vs. 7.4%, *p* = 0.181) and anticoagulant usage at discharge (9.9% vs. 10.3%, *p* = 0.635) between the IV rt‐PA group and the non‐reperfusion group (Table S3). At 1 year, the IV rt‐PA group versus the non‐reperfusion group had a significantly higher proportion of patients with functional independence (mRS 0–2: 70.9% vs. 66.4%; OR, 1.25 [95% CI, 1.12–1.39]) and favorable outcomes (mRS 0–1: 59.5% vs. 54.6%; OR, 1.23 [95% CI, 1.11–1.36]) (both *p* < 0.001) (Table 2 and Table S4). A lower proportion of severe disability/death was also observed in the IV rt‐PA group versus the non‐reperfusion group (mRS 5–6: 15.9% vs. 20.3%; OR, 0.73 [95% CI, 0.64–0.83]; *p* < 0.001) (Table 2 and Table S4). Patients in the IV rt‐PA group had significantly lower mRS scores compared with the non‐reperfusion group, with a median (IQR) mRS score of 1 (0–3) versus 1 (0–3) at 1 year (common OR, 0.85 [95% CI, 0.77–0.93]; *p* < 0.001). The distribution of mRS scores at 1 year in each group is shown in Figure 2. The propensity score weighting analysis that included the non‐matched cohort showed similar results with the above main findings (Table S5). Analysis to compare the baseline characteristics between the IV rt‐PA group and the non‐reperfusion group among patients included in the functional outcome analysis is shown in Table S6. Exploratory analyses on the primary and secondary endpoints for baseline NIHSS score, time from symptom onset to treatment, age, and rt‐PA dose were performed (Figure S2).

**TABLE 2:** One‐Year All‐Cause Mortality and Functional Outcomes in the Propensity Score‐Matched IV rt‐PA and Non‐Reperfusion Cohorts.

	IV rt‐PA (*N* = 3247)	Non‐reperfusion (*N* = 3247)	χ^2^ ^b^	*p*	HR or OR (95% CI)	*p*
All‐cause mortality	360/3247 (11.1)	396/3247 (12.2)	1.94	0.164	0.90 (0.78–1.05)^c^	0.183
Functional independence (mRS 0–2)^a^	2187/3083 (70.9)	2046/3083 (66.4)	14.98	< 0.001	1.25 (1.12–1.39)^d^	< 0.001
Favorable clinical outcomes (mRS 0–1)^a^	1834/3083 (59.5)	1682/3083 (54.6)	15.29	< 0.001	1.23 (1.11–1.36)^d^	< 0.001
Severe disability/death (mRS 5–6)^a^	489/3083 (15.9)	626/3083 (20.3)	20.55	< 0.001	0.73 (0.64–0.83)^d^	< 0.001

*Note:* All data presented as n/N (%) unless otherwise stated.

Abbreviations: CI, confidence interval; HR, hazard ratio; IV, intravenous; mRS, modified Rankin Scale; OR, odds ratio; rt‐PA, recombinant tissue plasminogen activator.

^a^ Data from patients with available mRS scores at 1 year (*N* = 3083).

^b^ χ^2^ and *p*‐values were derived from the chi‐squared test.

^c^ HR (95% CI) and *p*‐value for all‐cause mortality derived from the Cox proportional hazards models with stratification by matching pairs.

^d^ OR (95% CI) and *p*‐values for functional outcomes derived from conditional logistic regression with stratification by matching pairs.

The baseline patient demographics and clinical characteristics remained similar between the IV rt‐PA and non‐reperfusion groups after patients who received endovascular treatment were included for sensitivity analysis (Table S7). Consistent with the study outcomes reported in the main analyses, no significant difference was observed in 1‐year all‐cause mortality in the IV rt‐PA versus non‐reperfusion group (12.6% vs. 12.4%; HR, 1.02 [95% CI, 0.89–1.17]; *p* = 0.775) (Figure S3 and Table S8). A significantly greater proportion of those treated with IV rt‐PA reported favorable outcomes or functional independence compared with those in the non‐reperfusion group, and a smaller proportion reported severe disability or death at 1 year (Table S8). Patients in the IV rt‐PA cohort had lower mRS scores versus the non‐reperfusion cohort, with a median mRS score of 1(0–3) versus 1(0–3) (common OR, 0.88 [95% CI, 0.80–0.96]; *p* = 0.004) (Figure S4). Analysis to compare the baseline characteristics between the IV rt‐PA group and the non‐reperfusion group among patients included in the functional outcome analysis is shown in Table S9.

The post hoc analysis of all‐cause mortality at < 150 days and ≥ 150 days after the onset of AIS showed that the IV rt‐PA cohort had a significantly lower risk of mortality than the non‐reperfusion cohort at ≥ 150 days post‐treatment (2.6% vs. 4.0%; HR, 0.61 [95% CI, 0.45–0.83]; *p* = 0.002). There was no statistically significant difference between the two groups at < 150 days post‐treatment (8.6% vs. 8.4%; HR, 1.03 [95% CI, 0.86–1.22]; *p* = 0.758) (Table S10 and Figure S5).

## 4 Discussion

This analysis of a large multicenter stroke registry demonstrated that at 1 year, the all‐cause mortality was similar between patients treated with IV rt‐PA and those in the non‐reperfusion group in Chinese patients with AIS eligible for IV thrombolysis, who arrived or were admitted to the hospital within 4.5 h of symptom onset. Furthermore, more patients treated with IV rt‐PA had favorable outcomes or functional independence at 1 year compared with those who did not receive IV rt‐PA.

The benefits of IV rt‐PA administered within 4.5 h of symptom onset to patients with AIS are well established, with no difference in mortality risk at 3 months [5]. However, maintenance of the benefits of IV rt‐PA in the longer term is less established, especially the impact of IV rt‐PA on overall survival in patients with AIS. Previous randomized controlled trials with long‐term follow‐up of treatment with rt‐PA showed no difference in mortality rates up to 18 months post‐treatment compared with those who were not treated with IV rt‐PA, although these data were based on different thrombolysis eligibility criteria (3 h and 6 h) from current guidelines [13, 21]. Longer‐term follow‐up is challenging in randomized controlled trials; therefore, outcomes from extended monitoring duration would need to be drawn from real‐world studies reflecting routine clinical practices. Findings from large real‐world clinical registry analyses that included non‐Asian patients with AIS who received rt‐PA have found inconsistent results regarding the effect of IV rt‐PA on 1‐year or longer‐term (up to 3 years) mortality risk [14, 15, 16, 17, 18].

In the present study, the 1‐year all‐cause mortality rate was slightly lower for the IV rt‐PA group compared with the non‐reperfusion group, although the difference was not significant. Similarly, the open‐label IST‐3 trial (*n* = 1948) that assessed both acute and long‐term survival of patients treated with IV rt‐PA reported that the proportion of patients who received rt‐PA and died was non‐significantly lower than the standard‐care‐only group during the 3‐year follow‐up. However, further analysis revealed that while the mortality risk was higher for rt‐PA during the first 7 days of treatment, survival was significantly better among patients who survived between 8 days and 3 years (HR, 0.78 [95% CI, 0.68–0.90]) [14]. In this study, a crossover in the Kaplan–Meier survival curves at about 150 days was also observed; post hoc analysis revealed similar patterns whereby the risk of mortality was lower in the IV rt‐PA group than the non‐reperfusion group in the follow‐up beyond 150 days, up to 1 year after treatment. Together, these findings suggest the benefits of rt‐PA on better survival in the long term persist in patients who survive the initial risks of intracranial bleeding and death at the acute post‐thrombolysis phase [14]. In a propensity score‐matched analysis of a large registry study in South London, UK, with longer‐term follow‐up, IV rt‐PA was associated with a 28% decrease in mortality at 5 years (HR, 0.72 [95% CI, 0.60–0.87]) and a 37% decrease in mortality at 10 years (HR, 0.63 [95% CI, 0.48–0.82]), suggesting that treated patients lived an average of 1 year longer than controls [22]. The Kaplan–Meier estimates from the propensity score matching analysis of the nationwide South Korean registry (i.e., Asian population) also showed lower mortality at 5 years in patients who received thrombolysis with rt‐PA compared with the non‐intravenous thrombolysis group [23]. The observed delayed mortality benefit likely reflects cumulative indirect advantages of early functional recovery such as reduced bedridden‐related infections, cardiovascular complications, and other secondary consequences of prolonged disability [24, 25].

In the current study, we focused on the Chinese population and found that IV rt‐PA treatment was associated with improved 1‐year functional outcomes despite similar all‐cause mortality rates compared with non‐reperfusion patients. Our main findings were consistent in the sensitivity analysis, which included patients who received endovascular treatment, reflecting local clinical practice where AIS treatment may require both thrombolysis and endovascular treatment [26]. Similarly, in South Korea, a propensity score‐matched analysis of the nationwide South Korean registry with real‐world data showed that patients who received thrombolysis with rt‐PA had a lower mortality rate at 1 year (18.2% vs. 21.8%) and 5 years (31.5% vs. 38.6%) compared with the non‐intravenous thrombolysis group [23]. Our findings, along with those from South Korea, support the long‐term benefits of IV rt‐PA treatment for AIS in Asian populations. It is interesting to note that similar long‐term benefits of IV rt‐PA treatment have also been observed in European populations. A study from the South London Stroke Register reported that thrombolysis with intravenous alteplase was associated with improved long‐term survival and functional status at 5 years, suggesting that the long‐term benefits of IV rt‐PA treatment are not limited to Asian populations but are also evident in European populations [22]. Future studies should continue to explore these treatments in patients of different ethnicities in greater detail to optimize treatment strategies and improve patient outcomes.

Overall, these findings add evidence to suggest the beneficial effect of IV rt‐PA on long‐term survival. The mechanisms for how IV rt‐PA improves different factors that affect mortality remain unclear; however, previous studies have associated good functional outcomes with improved long‐term survival [17, 22]. In addition, patients with greater stroke severity were associated with a greater risk of death during the first 7 days post stroke [14]; acute myocardial infarction after AIS has also been associated with a threefold increase in in‐hospital mortality [27]. Rigorous implementation of stroke improvement and rehabilitation programs, as observed in the region [28], could have also contributed to better follow‐up and improved long‐term outcomes among patients who received intravenous thrombolytic treatment for AIS [16]. Importantly, early rehabilitation plays an essential role in avoiding long‐term disability [29].

This is the largest real‐world study in China that evaluated the 1‐year mortality risks between those who were treated with IV rt‐PA and those who did not receive reperfusion treatment using propensity score matching. The study analyzed data from the large CASE‐II registry, which continuously collected approximately 100,000 patient‐level data from a stroke quality control reporting system in hospitals in the Zhejiang province, and the population is representative of a developed province in eastern China. Compared with earlier thrombolysis studies, patients included in this study received IV rt‐PA within the treatment window that is recommended by the guidelines (within 4.5 h of symptom onset) and reflect a clinical population in routine practice where a diverse range of comorbidities is presented.

Several limitations should be considered when interpreting the study results. The registry did not collect detailed data on the reasons for each patient who did not receive reperfusion treatment, which may have resulted in potential selection bias or confounding effects during the analyses, although the use of propensity score matching and multivariate analysis would help to minimize potential sources of bias. Furthermore, this study did not include an analysis of recurrent stroke events or rehabilitation therapy between the IV rt‐PA group and the non‐reperfusion group, although there were no significant differences in any intracranial hemorrhage during hospitalization and anticoagulant usage at discharge between the IV rt‐PA group and the non‐reperfusion group. Finally, the patients in this study were slightly younger and had less severe stroke severity compared with stroke populations in real‐world registries elsewhere, which may affect the generalizability of the findings.

## 5 Conclusions

In routine practice, IV rt‐PA treatment in Chinese patients with AIS and eligible for thrombolysis was associated with improved 1‐year functional outcomes despite sharing a similar 1‐year all‐cause mortality as those who did not receive any reperfusion treatments.

## Author Contributions

Data collection: Wansi Zhong, Haidi Jin, Zhicai Chen, Yi Chen, Shenqiang Yan, and Haitao Hu. Database construction: Min Lou. Data organization: Rui Xue, Xiaoxian Gong, Yuhui Huang, and Wansi Zhong. Formal analysis: Rui Xue, Xiaoxian Gong, Yuhui Huang, Wansi Zhong, and Changzheng Yuan. Research design: Rui Xue, Xiaoxian Gong, Yuhui Huang, Changzheng Yuan, and Min Lou. Drafting of the manuscript: Rui Xue, Xiaoxian Gong, and Yuhui Huang. Critical review of the manuscript for important intellectual content: All authors. Supervision: Yi Chen, Shenqiang Yan, Changzheng Yuan, and Min Lou.

## Ethics Statement

This study was approved by the Second Affiliated Hospital of Zhejiang University Institutional Review Board. The study was designed and performed in accordance with the study protocol, the Declaration of Helsinki, the International Conference on Harmonization Harmonized Tripartite Guidelines for Good Clinical Practice, and applicable national and local regulations.

## Consent

The authors have nothing to report.

## Conflicts of Interest

The authors declare no conflicts of interest.

## Supporting information


Data S1.

## Data Availability

The data that support the findings of this study are available on request from the corresponding author. The data are not publicly available due to privacy or ethical restrictions.
