# Supplementary material for: One‐Year Outcomes After Intravenous Recombinant Tissue Plasminogen Activator for Ischemic Stroke: A Real‐World Study
Source: CNS Neurosci Ther. 2025 Aug 7;31(8):e70543. doi: 10.1111/cns.70543 (PMC12329421; doi:10.1111/cns.70543)
Supplement: Supplementary file 1 — Data S1. [file CNS-31-e70543-s001.docx]

**Supplementary Materials**

## Table S1. Baseline characteristics of the study population prior to propensity score matching

## **Table S2. Baseline characteristics of patients with and without data on mortality at 1year**

## **Table S3. Post-thrombolysis safety in the Propensity Score-Matched IV rt-PA and Non-Reperfusion Cohorts**

## Table S4. Functional outcomes in the propensity score-matched IV rt-PA and non-reperfusion cohorts after multiple imputation for missing data on mRS score at 1 year

## Table S5. **One-year all-cause mortality and functional outcomes of the whole study population after weighting by propensity score**

## Table S6. Baseline characteristics of the propensity score-matched IV rt-PA and non-reperfusion groups in the functional outcome analysis

## Table S7. Baseline characteristics of the propensity score-matched IV rt-PA and non-reperfusion groups after including patients who received endovascular treatment

## Table S8. One-year all-cause mortality and functional outcomes in the propensity score-matched IV rt-PA and non-reperfusion groups after including patients who received endovascular treatment

## **Table S9. Baseline characteristics of the propensity score-matched IV rt-PA and non-reperfusion groups after including patients who received endovascular treatment in the functional outcome analysis**

## Table S10. One-year all-cause mortality in the propensity score-matched IV rt-PA and non-reperfusion cohorts at <150 days or ≥150 days post-treatment

## Figure S1. Patient selection.

## Figure S2. All-cause mortality and functional outcomes at 1 year in different subgroups.

## Figure S3. One-year all-cause mortality in the propensity score-matched IV rt-PA and non-reperfusion groups after including patients who received endovascular treatment.

## Figure S4. Distribution of mRS scores in the propensity score-matched IV rt-PA and non-reperfusion groups at 1 year after including patients who received endovascular treatment.

## Figure S5. Kaplan–Meier curve for all-cause mortality at <150 days or ≥150 days post-treatment in the propensity score-matched IV rt-PA and non-reperfusion cohorts.

**Table S1. Baseline characteristics of the study population prior to propensity score matching.**

| **Variables** | **IV rt-PA**  **(N=8014)** | **Non-reperfusion**  **(N=4537)** | **ASD** |
| --- | --- | --- | --- |
| Age, mean (SD), years | 69.9 (12.3) | 70.9 (12.6) | 0.076 |
| Male, n (%) | 4835 (60.3) | 2675 (59.0) | 0.028 |
| Medical insurance status, n (%) |  |  | 0.148^a^ |
| Urban employee basic medical insurance | 2421 (30.2) | 1269 (28.0) |  |
| Urban resident basic medical insurance | 2429 (30.3) | 1563 (34.5) |  |
| New rural cooperative medical insurance | 1644 (20.5) | 1016 (22.4) |  |
| Other insurance | 743 (9.3) | 279 (6.1) |  |
| No insurance | 777 (9.7) | 410 (9.0) |  |
| Smoking status, n (%) |  |  | 0.073 |
| Never | 5668 (70.7) | 3131 (69.0) |  |
| Former | 431 (5.4) | 323 (7.1) |  |
| Current | 1915 (23.9) | 1083 (23.9) |  |
| Prior mRS score 0–1, n (%) | 7223 (90.1) | 4442 (97.9) | 0.332^a^ |
| NIHSS score, median (IQR) | 6 (3-12) | 2 (1-6) | 0.510^a^ |
| Time from symptom onset to hospital admission, mean (SD), min | 98.6 (56.7) | 161.1 (72.1) | 0.964^a^ |
| Time from symptom onset to treatment, mean (SD), min | 154.8 (56.6) | NA | NA |
| Time from hospital admission to treatment, mean (SD), min | 56.9 (28.9) | NA | NA |
| rt-PA dosage, n (%) |  |  | NA |
| Standard dosage | 6406 (92.7) | NA |  |
| Low dosage | 505 (7.3) | NA |  |
| Comorbidities, n (%) |  |  |  |
| Diabetes | 1265 (15.8) | 805 (17.7) | 0.052 |
| Coronary artery disease | 788 (9.8) | 344 (7.6) | 0.080 |
| Atrial fibrillation | 1488 (18.6) | 594 (13.1) | 0.150^a^ |
| Prior stroke/transient ischemic attack | 993 (12.4) | 1202 (26.5) | 0.362^a^ |
| Hypertension | 5212 (65.0) | 2985 (65.8) | 0.016 |
| Co-medication, n (%) |  |  |  |
| Antiplatelet | 1203 (15.0) | 751 (16.6) | 0.042 |
| Oral anticoagulation | 133 (1.7) | 125 (2.8) | 0.075 |
| Lipid lowering | 748 (9.3) | 523 (11.5) | 0.072 |
| Hospital level, n (%) |  |  | 0.064 |
| Grade 2 | 1846 (23.0) | 1170 (25.8) |  |
| Grade 3 | 6168 (77.0) | 3367 (74.2) |  |

^a^Absolute standardized difference (ASD) ≥0.1 was considered significant.

IV, intravenous; mRS, modified Rankin Scale; NA, not applicable; NIHSS, National Institutes of Health Stroke Scale; rt-PA, recombinant tissue plasminogen activator; SD, standard deviation; IQR, interquartile range.

**Table S2. Baseline characteristics of patients with and without data on mortality at 1year**

| **Variables** | **With data on mortality**  **(N=12551)** | **without data on mortality**  **N=4001)** | **ASD** |
| --- | --- | --- | --- |
| **Age, mean (SD), years** | **70.3 (12.4)** | **69.3 (13.1)** | **0.075** |
| **Male, n (%)** | **7510 (59.8)** | **2425 (60.6)** | **0.016** |
| **Medical insurance status, n (%)** |  |  | **0.053** |
| **Urban employee basic medical insurance** | **3690 (29.4)** | **1162 (29.0)** |  |
| **Urban resident basic medical insurance** | **3992 (31.8)** | **1227 (30.7)** |  |
| **New rural cooperative medical insurance** | **2660 (21.2)** | **830 (20.7)** |  |
| **Other insurance** | **1022 (8.1)** | **348 ( 8.7)** |  |
| **No insurance** | **1187 (9.5)** | **434 (10.8)** |  |
| **Smoking status, n (%)** |  |  | **0.012** |
| **Never** | **2998 (23.9)** | **972 (24.3)** |  |
| **Former** | **754 ( 6.0)** | **233 ( 5.8)** |  |
| **Current** | **8799 (70.1)** | **2796 (69.9)** |  |
| **Prior mRS score 0–1, n (%)** | **11665 (92.9)** | **3640 (91.0)** | **0.072** |
| **NIHSS score, median (IQR)** | **4 (2-10)** | **5 (2-11)** | **0.066** |
| **Time from symptom onset to hospital admission, mean (SD), min** | **121.2 (69.5)** | **121.2 (69.4)** | **<0.001** |
| **Time from symptom onset to treatment, mean (SD), min** | **154.8 (56.6)** | **154.5 (55.4)** | **0.005** |
| **Time from hospital admission to treatment, mean (SD), min** | **56.9 (28.9)** | **57.9 (30.2)** | **0.032** |
| **rt-PA dosage, n (%)** |  |  | **0.022** |
| **Standard dosage** | **6406 (92.7)** | **2125 (93.2)** |  |
| **Low dosage** | **505 (7.3)** | **154 (6.8)** |  |
| **Comorbidities, n (%)** |  |  |  |
| **Diabetes** | **2070 (16.5)** | **622 (15.5)** | **0.026** |
| **Coronary artery disease** | **1132 ( 9.0)** | **319 ( 8.0)** | **0.038** |
| **Atrial fibrillation** | **2082 (16.6)** | **659 (16.5)** | **0.003** |
| **Prior stroke/transient ischemic attack** | **2195 (17.5)** | **650 (16.2)** | **0.033** |
| **Hypertension** | **8197 (65.3)** | **2554 (63.8)** | **0.031** |
| **Co-medication, n (%)** |  |  |  |
| **Antiplatelet** | **1954 (15.6)** | **541 (13.5)** | **0.058** |
| **Oral anticoagulation** | **258 (2.1)** | **93 (2.3)** | **0.018** |
| **Lipid lowering** | **1271 (10.1)** | **358 (8.9)** | **0.040** |
| **Hospital level, n (%)** |  |  | **0.095** |
| **Grade 2** | **3016 (24.0)** | **1128 (28.2)** |  |
| **Grade 3** | **9535 (76.0)** | **2873 (71.8)** |  |

Absolute standardized difference (ASD) <0.1 (not significant) for all baseline characteristics. IV, intravenous; mRS, modified Rankin Scale; NA, not applicable; NIHSS, National Institutes of Health Stroke Scale; rt-PA, recombinant tissue plasminogen activator; SD, standard deviation; IQR, interquartile range

**Table S3. Post-thrombolysis safety in the Propensity Score-Matched IV rt-PA and Non-Reperfusion Cohorts**

|  | **IV rt-PA**  **(N=3247)** | **Non-Reperfusion**  **(N=3247)** | **χ^2a^** | ***P* Value^a^** |
| --- | --- | --- | --- | --- |
| Any intracranial hemorrhage during hospitalization | 270/3247 (8.3) | 240/3247 (7.4) | 1.92 | 0.181 |
| Anticoagulant usage at discharge | 272/2755 (9.9) | 328/3193 (10.3) | 0.26 | 0.635 |

All data presented as n/N (%) unless otherwise stated.

^a^ **χ**^2^ and P value were derived from the chi-squared test.

IV, intravenous; rt-PA, recombinant tissue plasminogen activator.

**Table S4. Functional Outcomes in the Propensity Score-Matched IV rt-PA and Non-Reperfusion Cohorts after multiple imputation for missing data on mRS score at 1 year**

| **Variables** | **OR (95% CI)** | ***P* Value** |
| --- | --- | --- |
| Functional independence (mRS 0–2)^a^ | 1.28 (1.14, 1.42) | <0.001 |
| Favourable clinical outcomes (mRS 0–1)^a^ | 1.25 (1.13, 1.39) | <0.001 |
| Severe disability/death (mRS 5–6)^a^ | 0.72 (0.63, 0.81) | <0.001 |
| Distribution of mRS score at 1 year^b^ | 0.83 (0.76, 0.91) | <0.001 |

^a^ OR (95% CI) and *P* value for these outcomes were derived from the conditional logistic regression models with stratification by matching pairs.

^b^ OR (95% CI) and *P* values for this outcome were derived from the ordinal logistic regression model and common OR was reported.

Missing mRS data at 1 year for the total matched cohort of 6,494 were imputed.

OR, odds ratio; CI, confidence interval; mRS, modified Rankin Scale.

**Table S5. One-year all-cause mortality and functional outcomes of the whole study population after weighting by propensity score**

|  | **IV rt-PA**  **(N=8014)** | **Non-Reperfusion**  **(N=4537)** | **HR or OR (95% CI)** | ***P* Value** |
| --- | --- | --- | --- | --- |
| All-cause mortality | 1331/8014 (16.6) | 511/4537 (11.3) | 0.94 (0.79, 1.10)^b^ | 0.434 |
| Functional independence (mRS 0–2)^a^ | 5164/7903 (65.3) | 2938/4363 (67.3) | 1.06 (1.05, 1.08)^c^ | <0.001 |
| Favourable clinical outcomes (mRS 0–1)^a^ | 4285/7903 (54.2) | 2386/4363 (54.7) | 1.07 (1.05, 1.09)^c^ | <0.001 |
| Severe disability/death  (mRS 5–6)^a^ | 1738/7903 (22.0) | 817/4363 (18.7) | 0.94 (0.92, 0.95)^c^ | <0.001 |
| Distribution of mRS score at 1 year | / | / | 0.79 (0.76, 0.83)^d^ | <0.001 |

All data presented as n/N (%) unless otherwise stated.

^a^Data from patients with available mRS scores at 1 year.

^b^HR (95% CI) and P value for all-cause mortality derived from the Cox proportional hazards models.

^c^OR (95% CI) and P value for functional outcomes derived from logistic regression.

^d^OR (95% CI) and P value for this outcome were derived from the ordinal logistic regression model and common OR was reported.

CI, confidence interval; HR, hazard ratio; IV, intravenous; OR, odds ratio; mRS, modified Rankin Scale; rt-PA, recombinant tissue plasminogen activator.

**Table S6. Baseline characteristics of the propensity score-matched IV rt-PA and non-reperfusion groups in the functional outcome analysis**

| Variables | **IV rt-PA**  **(N=3083)** | **Non-reperfusion  (N=3083)** | **ASD** |
| --- | --- | --- | --- |
| Age, mean (SD), years | 70.0 (11.5) | 69.8 (12.9) | 0.016 |
| Male, n (%) | 1853 (60.1) | 1844 (59.8) | 0.006 |
| **Medical insurance status, n (%)** |  |  | 0.045 |
| Urban employee basic medical insurance | 912 (29.6) | 904 (29.3) |  |
| Urban resident basic medical insurance | 988 (32.0) | 996 (32.3) |  |
| New rural cooperative medical insurance | 615 (19.9) | 656 (21.3) |  |
| Other insurance | 235 (7.6) | 225 (7.3) |  |
| No insurance | 333 (10.8) | 302 (9.8) |  |
| **Smoking status, n (%)** |  |  | 0.006 |
| Never | 764 (24.8) | 757 (24.6) |  |
| Former | 202 (6.6) | 200 (6.5) |  |
| Current | 2117 (68.7) | 2126 (69.0) |  |
| Prior mRS score 0–1, n (%) | 2996 (97.2) | 2998 (97.2) | 0.004 |
| NIHSS score, median (IQR) | 4 (2-8) | 3 (1-7) | 0.072 |
| Time from symptom onset to hospital admission, mean (SD), min | 133.1 (56.5) | 137.1 (68.7) | 0.064 |
| Time from symptom onset to treatment, mean (SD), min | 184.5 (54.3) | NA | NA |
| Time from hospital admission to treatment, mean (SD), min | 52.4 (26.2) | NA | NA |
| **rt-PA dosage, n (%)** |  |  | NA |
| Standard dosage | 2448 (92.2) | NA |  |
| Low dosage | 207 (7.8) | NA |  |
| **Comorbidities, n (%)** |  |  |  |
| Diabetes | 528 (17.1) | 526 (17.1) | 0.002 |
| Coronary artery disease | 243 (7.9) | 232 (7.5) | 0.013 |
| Atrial fibrillation | 412 (13.4) | 410 (13.3) | 0.002 |
| Prior stroke/transient ischemic attack | 567 (18.4) | 577 (18.7) | 0.008 |
| Hypertension | 2029 (65.8) | 1982 (64.3) | 0.032 |
| **Co-medication, n (%)** |  |  |  |
| Antiplatelet | 480 (15.6) | 472 (15.3) | 0.007 |
| Oral anticoagulation | 56 (1.8) | 70 (2.3) | 0.032 |
| Lipid lowering | 324 (10.5) | 319 (10.3) | 0.005 |
| **Hospital level, n (%)** |  |  | 0.005 |
| Grade 2 | 729 (23.6) | 736 (23.9) |  |
| Grade 3 | 2354 (76.4) | 2347 (76.1) |  |

Absolute standardized difference (ASD) <0.1 (not significant) for all baseline characteristics. IV, intravenous; mRS, modified Rankin Scale; NA, not applicable; NIHSS, National Institutes of Health Stroke Scale; rt-PA, recombinant tissue plasminogen activator; SD, standard deviation; IQR, interquartile range.

**Table S*7*. Baseline characteristics of the propensity score-matched IV rt-PA and non-reperfusion groups after including patients who received endovascular treatment**

| Variables | **IV rt-PA**  **(N=3328)** | **Non-reperfusion  (N=3328)** | **ASD** |
| --- | --- | --- | --- |
| Age, mean (SD), years | 70.2 (11.7) | 70.3 (12.9) | 0.009 |
| Male, n (%) | 1968 (59.1) | 1979 (59.5) | 0.007 |
| Medical insurance status, n (%) |  |  | 0.033 |
| Urban employee basic medical insurance | 989 (29.7) | 974 (29.3) |  |
| Urban resident basic medical insurance | 1059 (31.8) | 1088 (32.7) |  |
| New rural cooperative medical insurance | 698 (21.0) | 718 (21.6) |  |
| Other insurance | 242 (7.3) | 227 (6.8) |  |
| No insurance | 340 (10.2) | 321 (9.6) |  |
| Smoking status, n (%) |  |  | 0.005 |
| Never | 2310 (69.4) | 2310 (69.4) |  |
| Former | 206 (6.2) | 210 (6.3) |  |
| Current | 812 (24.4) | 808 (24.3) |  |
| Prior mRS score 0–1, n (%) | 3226 (96.9) | 3236 (97.2) | 0.018 |
| NIHSS score, median (IQR) | 4 (2-8) | 3 (1-8) | 0.043 |
| Time from symptom onset to hospital admission, mean (SD), min | 133.5 (56.3) | 137.4 (68.2) | 0.063 |
| Time from symptom onset to treatment, mean (SD), min | 184.9 (53.8) | NA | NA |
| Time from hospital admission to treatment, mean (SD), min | 52.4 (25.9) | NA | NA |
| rt-PA dosage, n (%) |  |  | NA |
| Standard dosage | 2637 (92.0) | NA |  |
| Low dosage | 229 (8.0) | NA |  |
| Comorbidities, n (%) |  |  |  |
| Diabetes | 570 (17.1) | 566 (17.0) | 0.003 |
| Coronary artery disease | 250 (7.5) | 251 (7.5) | 0.001 |
| Atrial fibrillation | 469 (14.1) | 470 (14.1) | 0.001 |
| Prior stroke/transient ischemic attack | 613 (18.4) | 645 (19.4) | 0.025 |
| Hypertension | 2174 (65.3) | 2159 (64.9) | 0.009 |
| Co-medication, n (%) |  |  |  |
| Antiplatelet | 506 (15.2) | 520 (15.6) | 0.012 |
| Oral anticoagulation | 72 (2.2) | 77 (2.3) | 0.010 |
| Lipid lowering | 330 (9.9) | 345 (10.4) | 0.015 |
| Hospital level, n (%) |  |  | 0.029 |
| Grade 2 | 740 (22.2) | 780 (23.4) |  |
| Grade 3 | 2588 (77.8) | 2548 (76.6) |  |
| Endovascular treatment, n (%) | 86 (2.6) | 90 (2.7) | 0.007 |

Absolute standardized difference (ASD) <0.1 (not significant) for all baseline characteristics. Endovascular treatment was used in propensity score matching. IV, intravenous; mRS, modified Rankin Scale; NA, not applicable; NIHSS, National Institutes of Health Stroke Scale; rt-PA, recombinant tissue plasminogen activator; SD, standard deviation; IQR, interquartile range.

## Table S8. One-year all-cause mortality and functional outcomes in the propensity score-matched IV rt-PA and non-reperfusion groups after including patients who received endovascular treatment

|  | **IV rt-PA** | **Non-reperfusion** | **χ^2b^** | ***P*-value^b^** | **HR or OR (95% CI)** | ***P*-value** |
| --- | --- | --- | --- | --- | --- | --- |
| All-cause mortality | 419/3328 (12.6) | 414/3328 (12.4) | 0.03 | 0.853 | 1.02 (0.89–1.17)^c^ | 0.775 |
| Functional independence (mRS 0–2)^a^ | 2194/3150 (69.7) | 2074/3150 (65.8) | 10.46 | 0.001 | 1.19 (1.07–1.32)^d^ | 0.001 |
| Favourable clinical outcomes (mRS 0–1)^a^ | 1838/3150 (58.3) | 1694/3150 (53.8) | 13.36 | <0.001 | 1.21 (1.09–1.34)^d^ | <0.001 |
| Severe disability/death (mars 5–6)^a^ | 563/3150 (17.9) | 656/3150 (20.8) | 8.80 | 0.003 | 0.82 (0.73–0.94)^d^ | 0.003 |

All data presented as n/N (%) unless otherwise stated.

^a^Data from patients with available mRS scores at 1 year (N = 3150).

^b^χ^2^ and P-values were derived from the chi-squared test.

^c^HR (95% CI) and P-value for all-cause mortality derived from the Cox proportional hazards models with stratification by matching pairs.

^d^OR (95% CI) and P-values for functional outcomes derived from conditional logistic regression.

CI, confidence interval; HR, hazard ratio; IV, intravenous; OR, odds ratio; mRS, modified Rankin Scale; rt-PA, recombinant tissue plasminogen activator.

## **Table S9. Baseline characteristics of the propensity score-matched IV rt-PA and non-reperfusion groups after including patients who received endovascular treatment in the functional outcome analysis**

| Variables | IV rt-PA  (N=3150) | Non-reperfusion  (N=3150) | ASD |
| --- | --- | --- | --- |
| Age, mean (SD), years | 70.1 (11.7) | 69.9 (12.9) | 0.017 |
| Male, n (%) | 1863 (59.1) | 1892 (60.1) | 0.019 |
| Medical insurance status, n (%) |  |  | 0.025 |
| Urban employee basic medical insurance | 941 (29.9) | 922 (29.3) |  |
| Urban resident basic medical insurance | 996 (31.6) | 1024 (32.5) |  |
| New rural cooperative medical insurance | 667 (21.2) | 676 (21.5) |  |
| Other insurance | 226 (7.2) | 216 (6.9) |  |
| No insurance | 320 (10.2) | 312 (9.9) |  |
| Smoking status, n (%) |  |  | 0.012 |
| Never | 774 (24.6) | 781 (24.8) |  |
| Former | 195 (6.2) | 203 ( 6.4) |  |
| Current | 2181 (69.2) | 2166 (68.8) |  |
| Prior mRS score 0–1, n (%) | 3059 (97.1) | 3064 (97.3) | 0.010 |
| NIHSS score, median (IQR) | 4 (2-8) | 3 (1-8) | 0.062 |
| Time from symptom onset to hospital admission, mean (SD), min | 133.6 (56.3) | 137.2 (68.3) | 0.058 |
| Time from symptom onset to treatment, mean (SD), min | 184.7 (54.0) | NA | NA |
| Time from hospital admission to treatment, mean (SD), min | 52.2 (25.6) | NA | NA |
| rt-PA dosage, n (%) |  |  | NA |
| Standard dosage | 2497 (92.1) | NA |  |
| Low dosage | 214 (7.9) | NA |  |
| Comorbidities, n (%) |  |  |  |
| Diabetes | 534 (17.0) | 534 (17.0) | <0.001 |
| Coronary artery disease | 231 (7.3) | 230 (7.3) | 0.001 |
| Atrial fibrillation | 432 (13.7) | 423 (13.4) | 0.008 |
| Prior stroke/transient ischemic attack | 566 (18.0) | 602 (19.1) | 0.029 |
| Hypertension | 2056 (65.3) | 2032 (64.5) | 0.016 |
| Co-medication, n (%) |  |  |  |
| Antiplatelet | 473 (15.0) | 485 (15.4) | 0.011 |
| Oral anticoagulation | 65 (2.1) | 71 (2.3) | 0.013 |
| Lipid lowering | 309 ( 9.8) | 323 (10.3) | 0.015 |
| Hospital level, n (%) |  |  | 0.028 |
| Grade 2 | 701 (22.3) | 738 (23.4) |  |
| Grade 3 | 2449 (77.7) | 2412 (76.6) |  |
| Endovascular treatment, n (%) | 81 (2.6) | 80 (2.5) | 0.002 |

Absolute standardized difference (ASD) <0.1 (not significant) for all baseline characteristics. IV, intravenous; mRS, modified Rankin Scale; NA, not applicable; NIHSS, National Institutes of Health Stroke Scale; rt-PA, recombinant tissue plasminogen activator; SD, standard deviation; IQR, interquartile range.

## Table S10. One-year all-cause mortality in the propensity score-matched IV rt-PA and non-reperfusion cohorts at <150 days or ≥150 days post-treatment.

| **All-cause mortality, n (%)** | **IV rt-PA**  **(N = 3247)** | **Non-reperfusion**  **(N = 3247)** | **χ^2b^** | ***P*-value^b^** | **HR (95% CI)^c^** | ***P*-value^c^** |
| --- | --- | --- | --- | --- | --- | --- |
| <150 days | 278 (8.6) | 272 (8.4) | 0.070 | 0.789 | 1.03 (0.86–1.22) | 0.758 |
| ≥150 days^a^ | 71 (2.6) | 110 (4.0) | 8.690 | 0.003 | 0.61 (0.45–0.83) | 0.002 |

^a^ N = 2730 for each treatment group.

^b^χ^2^ and P-values were derived from the chi-squared test.

^c^HR (95% CI) and P-values were derived from Cox regression with stratification by matching pairs.

CI, confidence interval; HR, hazard ratio; IV, intravenous; rt-PA, recombinant tissue plasminogen activator.

## Figure S1. Patient selection.


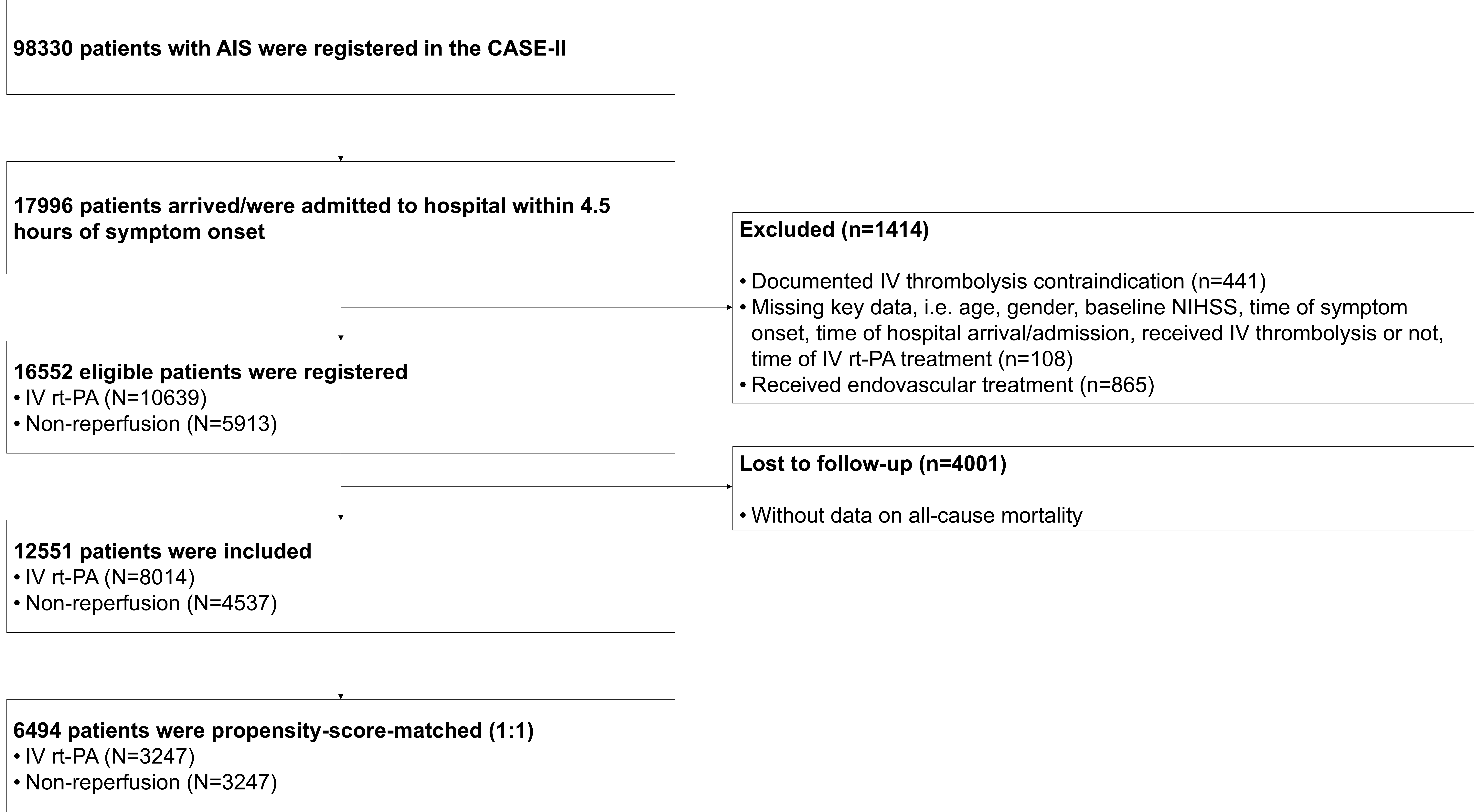


AIS, acute ischemic stroke; CASE-II, Computer-based Online Database of Acute Stroke Patients for Stroke Management Quality Evaluation; IV, intravenous; NIHSS, National Institutes of Health Stroke Scale; rt-PA, recombinant tissue plasminogen activator.

## Figure S2. All-cause mortality and functional outcomes at 1 year in different subgroups.


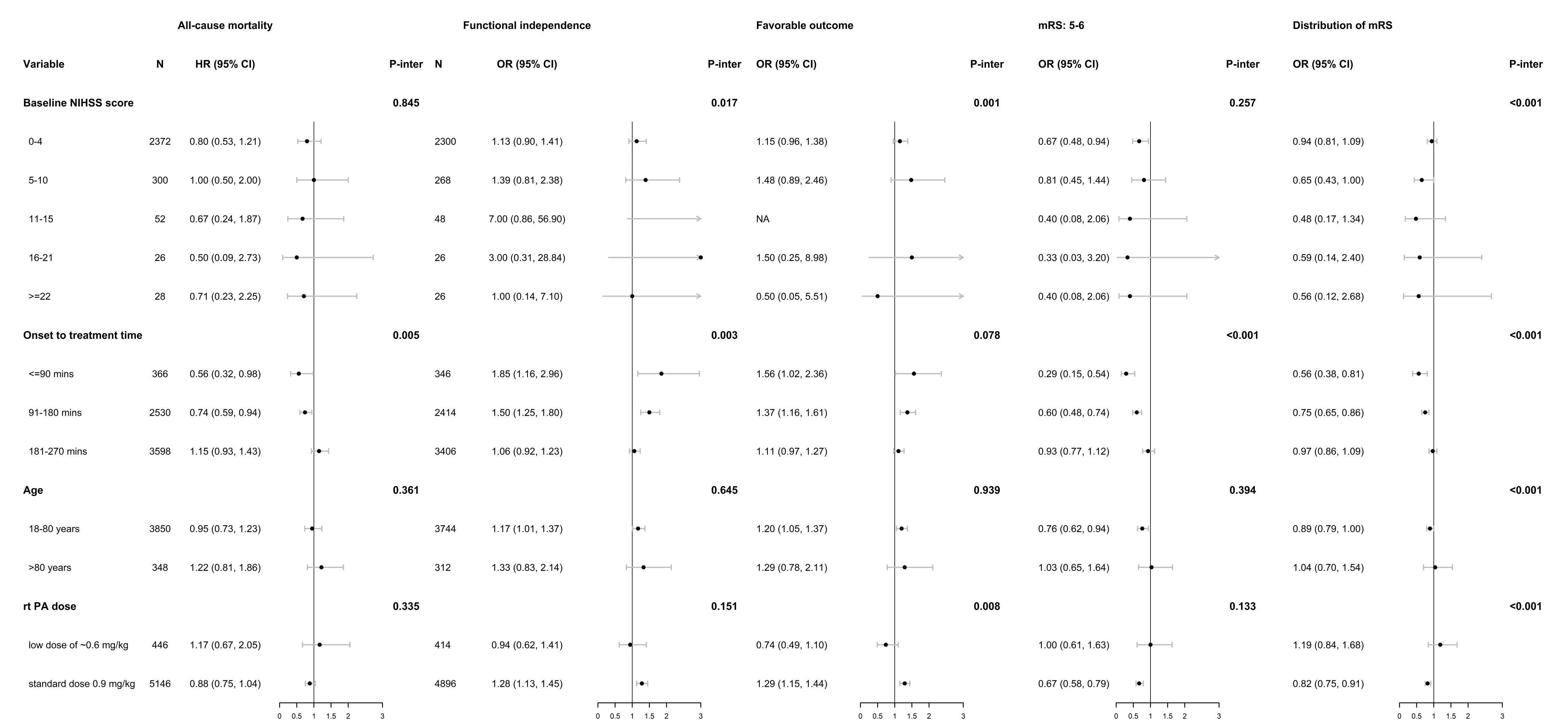


CI, confidence interval; HR, hazard ratio; mRS, modified Rankin Scale; NIHSS, National Institutes of Health Stroke Scale; OR, odds ratio; rt-PA, recombinant tissue plasminogen activator. The distribution of mRS at 1 year was analyzed using the common OR. The P-inter denote P for interaction. The first column of "No" represents the sample size for the primary outcome analysis, while the second column of "No" represents the sample size for the functional outcome analysis. Only matching pairs that both belong to the same subgroup are included in the analysis of that subgroup.

## Figure S3. One-year all-cause mortality in the propensity score-matched IV rt-PA and non-reperfusion groups after including patients who received endovascular treatment.


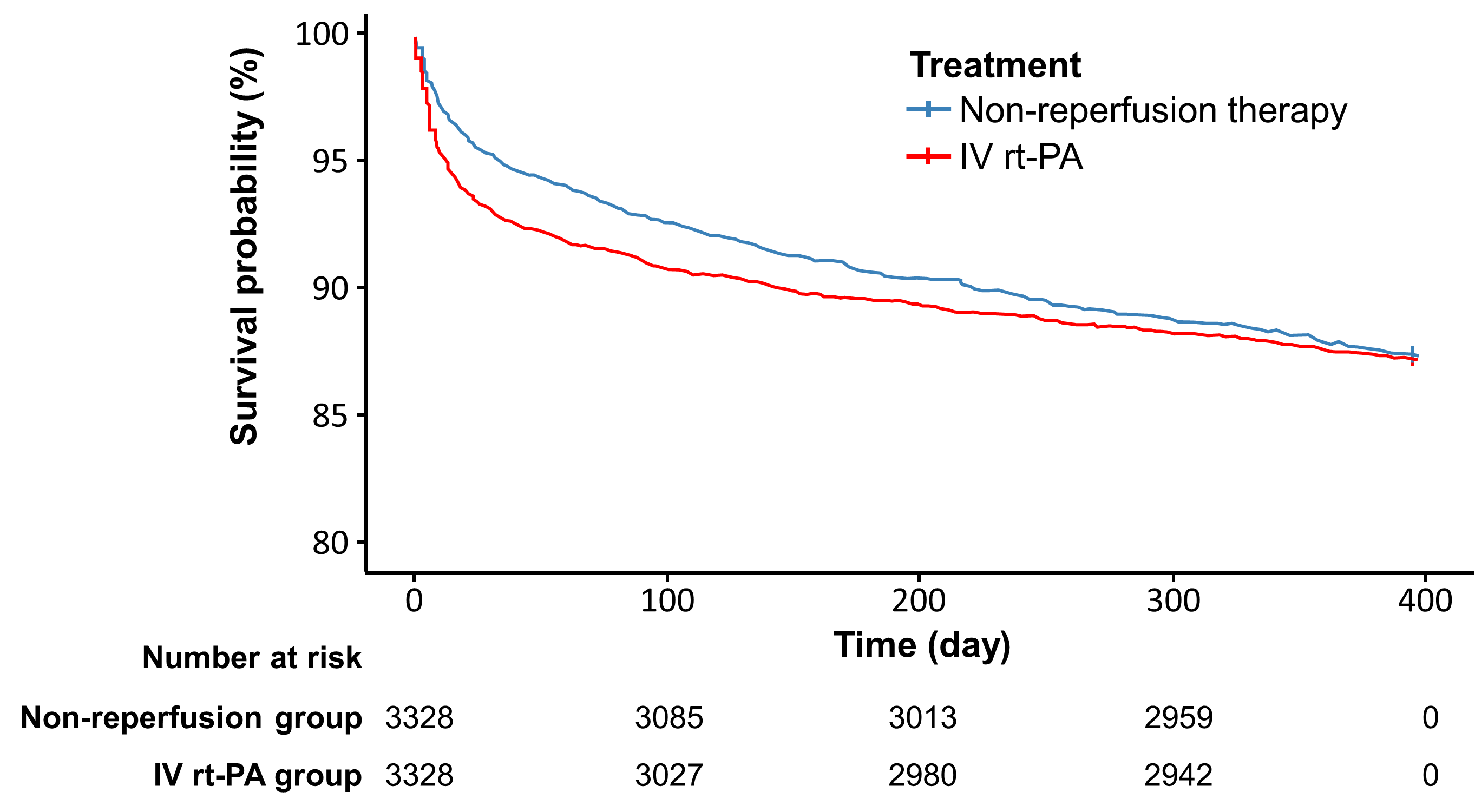


IV, intravenous; rt-PA, recombinant tissue plasminogen activator.

## Figure S4. Distribution of mRS scores in the propensity score-matched IV rt-PA and non-reperfusion groups at 1 year after including patients who received endovascular treatment.


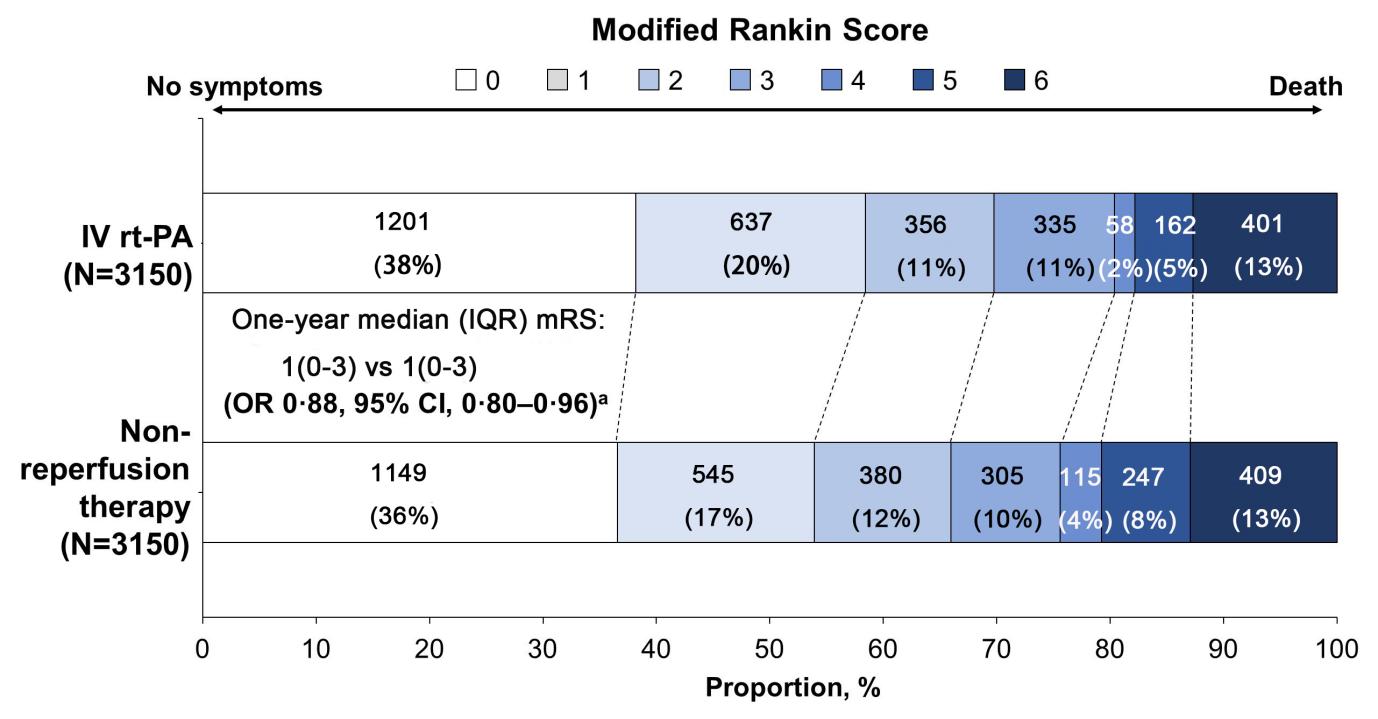


Data from patients with available mRS scores at 1 year (N = 3150). ^a^P = 0.004, derived from ordinal logistic regression. CI, confidence interval; IV, intravenous; mRS, modified Rankin Scale; OR, common odds ratio; rt-PA, recombinant tissue plasminogen activator; IQR, interquartile range.

## Figure S5. Kaplan–Meier curve for all-cause mortality at <150 days or ≥150 days post-treatment in the propensity score-matched IV rt-PA and non-reperfusion cohorts.


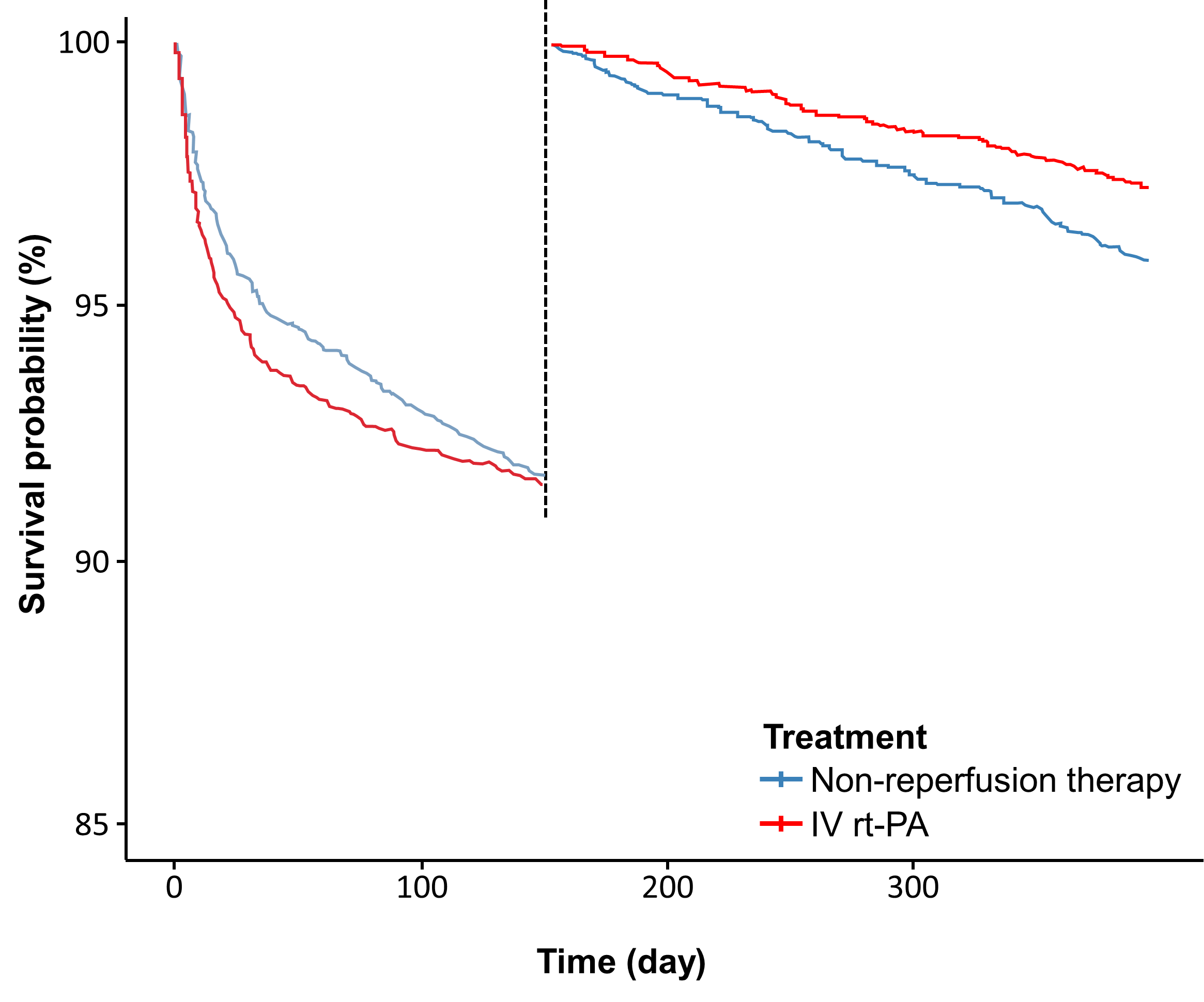


IV, intravenous; rt-PA, recombinant tissue plasminogen activator.
